# Depsidone Derivatives and a Cyclopeptide Produced by Marine Fungus *Aspergillus unguis* under Chemical Induction and by Its Plasma Induced Mutant

**DOI:** 10.3390/molecules23092245

**Published:** 2018-09-03

**Authors:** Wen-Cong Yang, Hai-Yan Bao, Ya-Yue Liu, Ying-Ying Nie, Jing-Ming Yang, Peng-Zhi Hong, Yi Zhang

**Affiliations:** 1 Research Institute for Marine Drugs and Nutrition, College of Food Science and Technology, Guangdong Provincial Key Laboratory of Aquatic Product Processing and Safety, Guangdong Province Engineering Laboratory for Marine Biological Products, Guangdong Ocean University, Zhanjiang 524088, China; yangwc_1993@163.com (W.-C.Y.); baohaiyanbhy@163.com (H.-Y.B.); yayue_liu@163.com (Y.-Y.L.); hdyjynyy502@163.com (Y.-Y.N.); m13266410079@163.com (J.-M.Y.); hongpengzhi@126.com (P.-Z.H.); 2 School of Environmental and Chemical Engineering, Dalian Jiaotong University, Dalian 116028, China; 3 Shenzhen Institute of Guangdong Ocean University, Shenzhen 518120, China

**Keywords:** depsidones, *Aspergillus unguis*, chemical induction, plasma-induced mutant, docking

## Abstract

A new depsidone derivative (**1**), aspergillusidone G, was isolated from a marine fungus *Aspergillus unguis*, together with eight known depsidones (**2**‒**9**) and a cyclic peptide (**10**): agonodepside A (**2**), nornidulin (**3**), nidulin (**4**), aspergillusidone F (**5**), unguinol (**6**), aspergillusidone C (**7**), 2-chlorounguinol (**8**), aspergillusidone A (**9**), and unguisin A (**10**). Compounds **1**‒**4** and **7**‒**9** were obtained from the plasma induced mutant of this fungus, while **5**, **6**, and **10** were isolated from the original strain under chemical induction. Their structures were identified using spectroscopic analysis, as well as by comparison with literature data. The HPLC fingerprint analysis indicates that chemical induction and plasma mutagenesis effectively influenced the secondary metabolism, which may be due to their regulation in the key steps in depsidone biosynthesis. In bioassays, compound **9** inhibited acetylcholinesterase (AChE) with IC_50_ in 56.75 μM. Compounds **1**, **5**, **7**, **8**, and **9** showed moderate to strong activity towards different microbes. Compounds **3**, **4**, and **5** exhibited potent larvicidality against brine shrimp. In docking studies, higher negative CDOCKER interaction energy and richer strong interactions between AChE and **9** explained the greater activity of **9** compared to **1**. Chemical induction and plasma mutagenesis can be used as tools to expand the chemodiversity of fungi and obtain useful natural products.

## 1. Introduction

**Figure 1:**
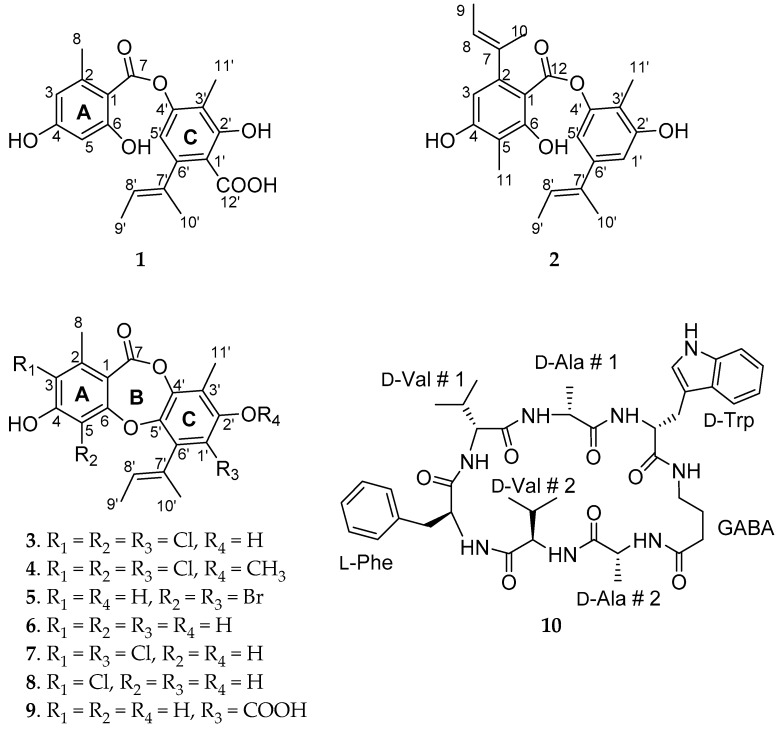
Structures of compounds **1**‒**10**.

Metabolites from marine fungi are substantial source for drug discovery. The unique structures produced by marine fungi are highly valued in the development of clinical drugs, such as antitumor, anti-HIV, antifungal, antibacterial, anti-acetylcholinesterase, and antioxidant drugs [1,2,3,4]. However, the compounds which have been reported are only a tiny part of the fungal secondary metabolic potential, arousing researchers’ general interest in activating silence genes in the research of microbial natural products. There are many methods in the application of this, such as epigenetic modifiers, plasma treatment, genome mining, and the “one strain many compounds” (OSMAC) methods [5,6,7,8,9]. Chemical induction has been attractive for its ability to upregulate the expression of secondary metabolic genes and increase chemodiversity, which has been demonstrated by the application of epigenetic remodeling methods with DNA methyltransferase and histone deacetylase inhibitors [10,11]. Plasma refers to an ionized gas-like physical state consisting of highly electrified nuclei and free electrons. It is a highly excited, unstable state, which contains a variety of physical and chemical mutagenic factors [12]. There has been some research regarding *Fusarium graminearum* and *Klebsiella pneumoniae* treatment with plasma to change their metabolites [13,14]. However, there is no report about using the combination of epigenetic modifications with halogenated stimuli and treating marine fungi with plasma in natural product research. Depsidones are typical metabolites of *Aspergillus unguis* and other species in this genus. They usually show good potential as antitumor, antimicrobial, and insecticidal reagents [15]. Therefore, in this paper, two strategies—namely the use of a plasma-induced mutant and the combined use of epigenetic modifier (procaine, a DNA methyltransferase inhibitor) with sodium bromide—were employed to investigate their influence on the secondary metabolism of a marine strain of *A. unguis.* The aim is to produce new metabolites and measure their biological activities. The structures of a new depsidone derivative (**1**) together with eight known depsidones (**2**‒**9**) and a cyclic peptide (**10**) isolated from *A. unguis* under chemical induction and by its plasma-induced mutant are shown in Figure 1. Herein, we report the structure elucidation of the new compound, its secondary metabolism variation under regulations, and the bioactivity of all the compounds.

## 2. Results and Discussion

**Figure 2:**
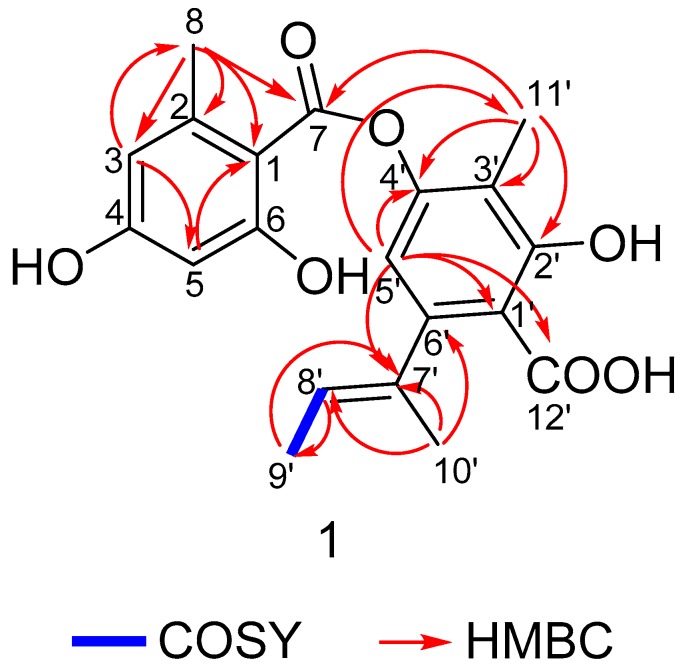
Key COSY (bold line) and HMBC (arrow) correlations of compound **1**.

Compound (**1**) was isolated as a yellow amorphous solid, m.p. 166.4‒167.6 °C. Its molecular formula was determined as C_20_H_20_O_7_ on the basis of the analysis of HR-ESI-MS at *m*/*z* 371.1137 [M − H]^−^ (calcd 371.1131) and NMR spectral data (see Table 1 and Supplementary Materials). It exhibited IR absorptions at 1658 and 3255 cm^−1^, suggesting the presence of ester carbonyl and hydroxyl moieties. Its UV (MeOH) λmax (log *ε*) was 217 (5.88), 266 (5.51), and 305 (5.27). The ^1^H-NMR spectrum showed the resonances of four methyls at *δ*_H_ 1.70 (3H, dd, *J* = 6.7, 0.8 Hz), 1.92 (3H, s), 2.01 (3H, s), and 2.59 (3H, s) and an olefinic and three aromatic protons groups were at *δ*_H_ 5.34 (1H, dq, *J* = 6.8, 1.3 Hz), 6.19 (1H, d, *J* = 2.4 Hz), 6.25 (1H, s), and 6.28 (1H, dd, *J* = 2.0, 0.5 Hz). The ^13^C-NMR spectrum exhibited a total of 20 carbon resonances, containing 12 aromatic carbons, 1 ester carbonyl, 1 carboxyl, 4 methyl groups, 1 tertiary carbon, and 1 quaternary carbon for aromatic rings, and 1-methyl-1-propenyl moiety, which accounted for all 11 degrees of unsaturation. As we know, ^1^H–^1^H couplings substantially reduce intensities of the C–H correlation. Therefore, it is hard or impossible to observe ^n^*J*_CH_ correlations for n > 3. However, the longer range ^n^*J*_CH_ couplings could be observed for some compounds with few or no ^1^H–^1^H couplings like depsidones, which can reach ^4^*J*_CH_, ^5^*J*_CH_, and ^7^*J*_CH_ couplings [16,17]. The HMBC correlations from H-8 to C-1, C-2, C-3, and C-7 (^4^*J*_CH_); from H-3 to C-1, C-2, and C-5; and from H-5 to C-1 established a 2-methyl-4,6-dihydroxyl-benzoyl moiety (aromatic ring A). The key HMBC correlation from H-11′ to C-2′, C-3′, and C-4′ and from H-5′ to C-1′, C-2′ (^4^*J*_CH_), C-3′, C-4′, C-7′, and C-12′ (COOH) (^4^*J*_CH_) created a 2-hydroxy-3-methyl-4-oxygenated group-6-acyl-benzoic acid moiety (aromatic ring C). The HMBC correlations from H-5′ to C-7 and from H-10′ to C-6′, C-7′ and C-8′, together with the ^1^H–^1^H COSY correlations from H-8′ to H-9′, established a moiety of 1-methyl-1-propenyl and attached it to C-6′ of ring C. Finally, the two rings were connected by the HMBC correlation from H-11′ to C-7(^5^*J*_CH_) to determine its structure (Figure 2). As for the configuration of the 2-but-2-enyl group, it has been observed by Beach and Richards that *O*-methylisonidulin (*trans*-configuration) has a higher splitting constant than nidulin (*cis*-configuration) for the interaction between the two vinyl methyls across the double bond [18]. Compound **1** possesses a splitting constant of 0.8 Hz close to nidulin with a splitting constant of 1.0 Hz, indicating that the 2-but-2-enyl group in **1** is assigned to be *cis*-configuration. Therefore, the structure of **1** was determined to be (*E*)-6-(but-2-en-2-yl)-4-((2,4-dihydroxy-6-methylbenzoyl)oxy)-2-hydroxy-3-methylbenzoic acid, named aspergillusidone G. This is the first time this compound has been reported. Structurally, it is a ring B-opened derivative of aspergillusidone A (compound **9**) [19].

The structures of compounds **2**‒**10** were identified as agonodepside A (**2**), nornidulin (**3**), nidulin (**4**), aspergillusidone F (**5**), unguinol (**6**), aspergillusidone C (**7**), 2-chlorounguinol (**8**), aspergillusidone A (**9**), and unguisin A (**10**), respectively, by comparison of their NMR data, MS, CD, and optical rotation with those in the literature [19,20,21,22,23,24,25].

**Figure 3:**
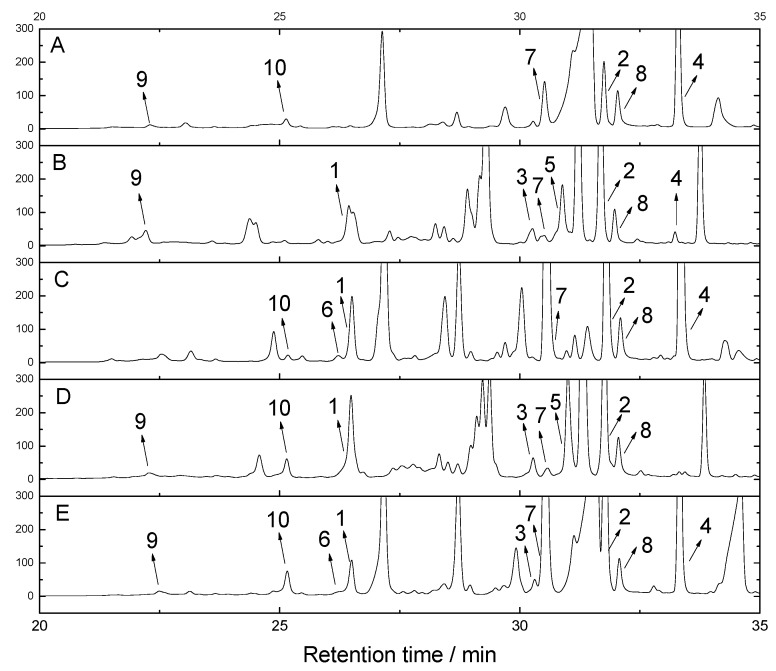
The HPLC fingerprints recorded at 254 nm of *Aspergillus unguis* fermentation extracts when cultured under different conditions. (**A**) *A. unguis* in potato sucrose broth (PSB) medium; (**B**) *A. unguis* in PSB medium + NaBr; (**C**) *A. unguis* in PSB medium + procaine; (**D**) *A. unguis* in PSB medium + procaine + NaBr; (**E**) *A. unguis* treat with plasma in PSB medium.

To analyze the influence of different factors on secondary metabolites production, a parallel fermentation experiment was performed. As the results, the HPLC fingerprints varied depending on the factors (culture medium or mutation) (Figure 3). For compounds **1**‒**10**, some significant differences were observed as following. Compound **1** was not detected in the extract of original strain cultured in normal PSB medium, while it was dramatically produced by the original strain cultured in PSB medium supplemented with NaBr, procaine, and especially with NaBr plus procaine. The yield of compound **2** was sharply increased in all the treatment groups. Compound **5** is a brominated depsidone, which was not produced or produced only a minute yield with the original strain in normal PSB and PSB with procaine. However, it was remarkably produced in PSB with NaBr and especially with NaBr plus procaine. This phenomenon inferred that NaBr and procaine showed synergistic effects on the production of compounds **1** and **5**. Intriguingly, compound **1** was also presented with higher yields by the mutant strain 6-20-6 in normal PSB medium. Compounds **4** and **7** both showed high yield only with the procaine-induced original strain and the mutant strain. The cyclopeptide **10** also presented higher yield under double chemical induction or with the mutant strain.

**Figure 4:**
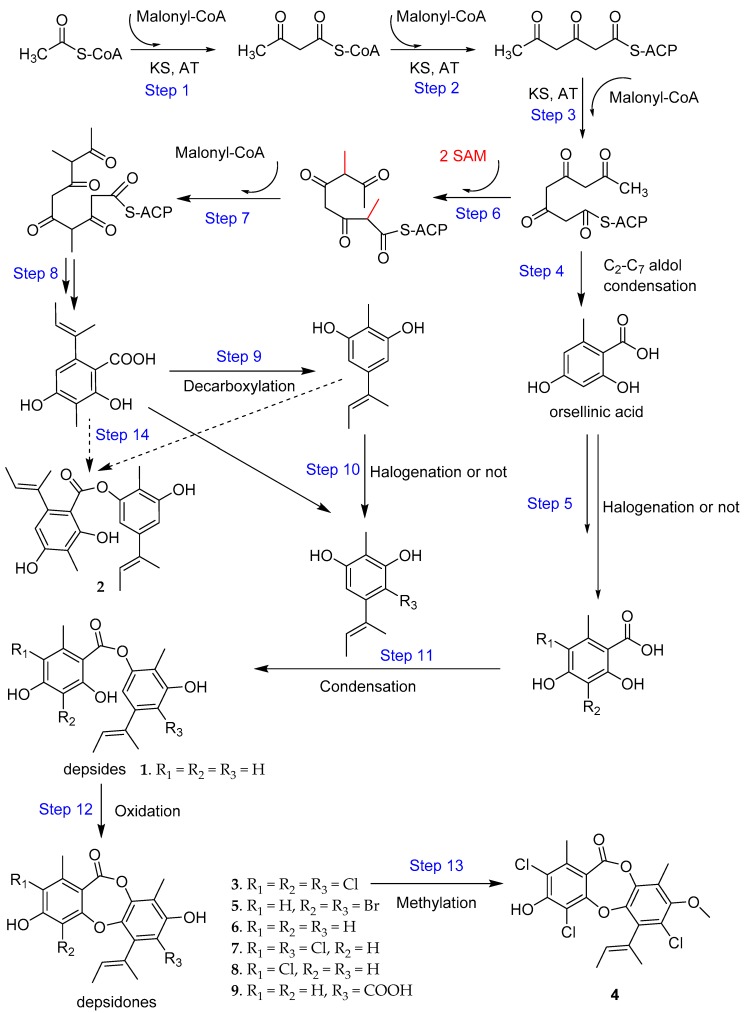
Probable proposed biosynthetic pathways for depsides **1**‒**2** and depsidones **3**‒**9**, KS, ketosynthase; AT, acytransferase; ACP, acyl carrier protein; SAM, S-Adenosyl methionione [19,26,27,28].

The biosynthetic pathways for fungal depsidones have already been described by Sureram, Sierankiewicz, Schümann, and Okoye [19,26,27,28]. The depsidones of fungus *A. unguis* were proposed to be biosynthesized via depside formation from orsellinic acid and orcinol derivatives, which were structurally diverse and derived from PKS pathways and post-PKS modifications. The possible biosynthetic pathways of compounds **1**‒**9** were presented based on previous proposals (Figure 4). Combining the biosynthetic pathways with the HPLC fingerprints, the possible mechanisms were analyzed for the dramatically enhanced production of compounds **1**, **2**, **4**, and **7** by the fungus when treated with procaine or plasma. It was observed that these two factors can upregulate the upstream steps 5, 10, 11, and others to yield more of compounds **1**, **4**, and **7**. They can also upregulate the steps 8, 9, and 14 to promote production of compound **2** (Figure 3 and Figure 4). Considering that the increases of compounds **1** and **2** are noncompetitive, the upregulation of steps 8, 9, and 10 should be substantial to provide sufficient orcinol precursors. The enhanced production of the bromide **5** and inhibited yield of chlorides **4** and **7** in NaBr treated groups were attributed the bromination–chlorination competition during steps 5 and 10. Since NaBr addition also promoted the yield of **1** and **2**, it should also affect production via the upstream steps as procaine and plasma do. 

Compounds **1**‒**10** were assayed for their antimicrobial activity against pathogenetic strains *Pseudomonas aeruginosa*, Methicillin-resistant *Staphylococcus aureus*, *Vibrio parahemolyticus*, and *Candida albicans*, with inhibition zones ranging from 6.0 to 17.7 mm at a dosage of 10 μg per disc (Table 2, each diameter of the paper disc is 6.0 mm). Compound **9** showed strong activity against *P. aeruginosa*, MRSA, and *C. albicans* at concentrations of 10 μg per disc. All compounds were rescreened using the microdilution method. As shown in Table 3, compounds **1** and **5**‒**9** showed moderate inhibition with minimal inhibitory concentration (MIC) values in the range of 6.4‒102.4 μM against *P. aeruginosa*. Compounds **1**, **4**‒**5**, and **8**‒**9** also achieved moderate inhibition with MIC values ranging from 25.6 to 102.4 μM to resist MRSA. Compounds **1** and **9** can inhibit *C. albicans* with MICs of 12.8 and 6.4 μM, respectively. However, there was no compound that could totally inhibit *V. parahemolyticus* within a concentration of 102.4 μM. Analysis of the structure–activity relationships of compounds **1**‒**9** revealed that only those molecules with one to two halogenated or carboxyl groups exhibited antifungal or antibacterial activities. A carboxyl group on ring C may be crucial to antifungal activity.

In addition, compounds **1**‒**10** were evaluated for their in vitro inhibitory activities to AChE, antioxidant activity using DPPH free radicals, and larvicidality using a brine shrimp test (Table 4). The results indicated all compounds except compound **9** displayed no or weak activity against AChE, with IC_50_ values exceeding 102.4 μM. Meanwhile, all compounds only exhibited a weak antioxidant activity, with an EC_50_ value over 102.4 μM. Compounds **3**‒**5** demonstrated strong larvicidal activity with close or even lower LC_50_ values compared with the positive control Hg(NO_3_)_2_. 

**Figure 5:**
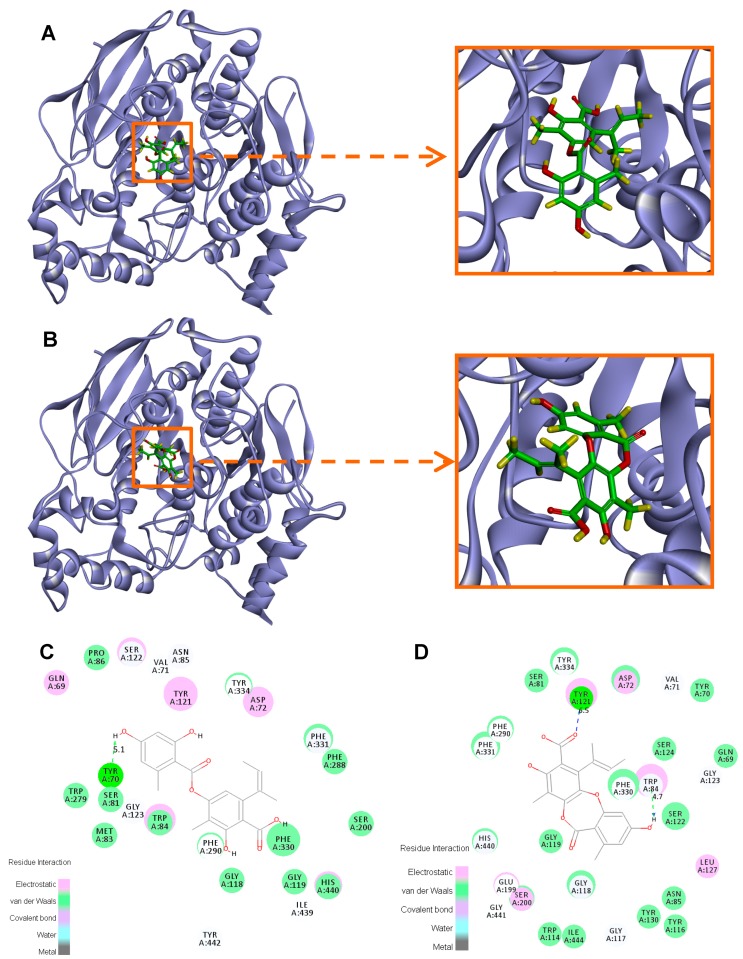
Representation of the binding mode of compounds **1** (**A**,**C**) and **9** (**B**,**D**) in the active site of AChE.

To explain the different potency of highly similar compounds **1** and **9** towards AChE, a docking study was performed using Discovery Studio 3.5 software (BIOVIA Co., Ltd., San Diego, CA, USA). Docking results showed that the docked pose of **1** with AChE has a negative CDOCKER energy of 10.02 kcal/mol and a negative CDOCKER interaction energy of 44.43 kcal/mol. It mainly formed one hydrogen bond with AChE, i.e., the one between 4-OH and Tyr 70 (bond length 5.1 Å) (Figure 5A,C). Electrostatic (Tyr 121, Ser 122, Trp 84, etc.) and van der Waals (Trp 84, Tyr 334, Phe 331, Phe 330, etc.) interactions exist between **1** and AChE. As for compound **9**, its docked pose with AChE has a negative CDOCKER energy of −7.86 kcal/mol and a negative CDOCKER interaction energy of 46.07 kcal/mol. Compound **9** formed two hydrogen bonds between its 4-OH and Trp 84 (4.7 Å) and between its 1′-COOH and Tyr 121 (6.5 Å) (Figure 5B,D). Besides, electrostatic (Tyr 121, Trp 84, etc.) and van der Waals (Tyr 334, Phe 331, Phe 330, Ser 122, etc.) interactions also exist between **9** and AChE. All these interactions are known to be formed between the potent AChE inhibitors and the enzyme active site [29,30,31,32]. In comparison, compound **9** possesses a higher negative CDOCKER interaction energy and stronger interactions with different sites within the AChE active site than **1**, which explains its higher activity. 

Depsidones are mainly produced by lichens and fungi. They are widely reported with antitumor, antimicrobial, antiviral, pesticidal, and other activities [33,34]. Some depsidones also show activity towards the central neural system. Two lichen derived depsidones, perlatolic acid and an acetylated artifact, were ever reported to be AChE inhibitors [35,36]. Here, we first report depsidone as an AChE inhibitor from marine fungi. 

## 3. Materials and Methods

### 3.1. General Experimental Procedures

1D and 2D NMR spectra were recorded on a Bruker Advance 500 MHz NMR spectrometer (Billerica, MA, USA) using tetramethylsilane as an internal standard. HR-ESI-MS spectra were obtained from a Burker maXis Q-TOF mass spectrometer (Billerica, MA, USA). UV-vis spectra were measured on a Shimadzu UV-2550 spectrometer (Kyoto, Japan). IR spectra were measured on a Bruker TENSOR 27 FT-IR spectrometer (Billerica, MA, USA). Optical rotations were measured on an Anton-Paar MCP500 automatic polarimeter (Graz, Austria) at room temperature. CD spectra were measured on an Applied Photophysics Chirascan spectropolarimeter (Surrey, UK). Melting points were tested on a WRX-4 melting point apparatus manufactured by YiCe Apparatus & Equipment Co., Ltd. (Shanghai, China). The HPLC fingerprints of *Aspergillus unguis* extracts were detected using an Agilent 1260 Infinity II (Palo Alto, CA, USA) with a DAD detector and a reversed-phased column (Agilent-packed EC.C18, 4 μm, 4.6 × 250 mm). A 96-well microplate reader manufactured by BioTek Epoch2 (Winooski, VT, USA) was used for all spectrophotometric measurements. A Büchi R-300 Rotavapor (Flawil, Switzerland) and a Büchi P-12 multivapor (Flawil, Switzerland) were used for sample condensation. Preparative HPLC chromatography was performed on a HP Plus 50D separation module coupled with a UV-vis detector manufactured by Lisure Co., Ltd. (Suzhou, China) and a preparative reversed-phased column (Elite-packed SinoChrom ODS-AP, 15 μm, 20.0 × 250 mm) was used for purification. Materials for column chromatography (CC) involved silica gel (60‒100, 100‒200, and 200‒300 mesh, Qingdao Marine Chemistry Co. Ltd., Qingdao, China), ODS gel (50 μm, YMC, Kyoto, Japan) and Sephadex LH-20 (18‒110 μm, GE Healthcare Bio-sciences AB, Stockholm, Sweden). Precoated silica gel plates (Merck, Silica gel 60 F254, Darmstadt, Germany) were used for TLC analysis. NKA resin (H&E Co., Ltd., Beijing, China) was used to absorb the metabolites in the broth.

### 3.2. Fungal Material and Fermentation

The fungal *A. unguis* DLEP2008001 was isolated from seaweed collected in Dalian, China, identified using ITS gene sequencing, and deposited in China General Microbiological Culture Collection Center labeled with the number of CGMCC 3372 as previously reported [15]. The fungus was activated and cultured on a PDA petri dish at 28 °C for four days. The spores were collected through washing with normal saline and were used as inoculum. The chemical induced fermentation was carried out in 20 × 3 L-Erlenmeyer flasks, each containing 1 L NaBr-PSP (each containing 20 g NaBr, 20 g sea salt, 20 g sucrose, 5 g peptone, and 500 mL potato juice). The contents were autoclaved at 121 °C for 15 min. After cooling to room temperature, each flask was inoculated with 5 mL of the spore inoculum and incubated at 28 °C for three days. Then, procaine was added to a final concentration of 1 mM, and the fungus was continuously incubated the fungus for 20 days. 

The fungal mutant 6-20-6 (*A. unguis* 6-20-6 GDMCC 60337) was obtained from the original strain *A. unguis* DLEP2008001 CGMCC 3372 using low temperature plasma treatment under atmospheric pressure [37]. Its taxonomical taq ITS rDNA gene sequence (GenBank accession number MH071299) showed no difference with that of the original strain. This mutant strain was fermented for 10 L following the same method as the original strain except that the liquid culture medium was normal PSB.

### 3.3. Extraction and Isolation

After filtration, the fermentation broth of the original strain under chemical induction was extracted three times with EtOAc (3 × 10 L). The mycelium was extracted three times with methanol. The two extracts were evaporated below 45 °C under a vacuum and combined to afford a dry crude extract (55 g). The crude extract was subjected to silica gel vacuum liquid chromatography (VLC) eluted with petroleum ether-chloroform (1:3‒0:1) and chloroform-methanol (100:1‒0:1) to afford eight fractions (Fr1‒Fr8). From Fr3 (8.5880 g), compound **5** (39.6 mg) was obtained through column chromatography on silica gel (petroleum ether-acetone, 5:1), Sephadex LH-20 (methanol), and preparative HPLC on silica gel (petroleum ether-chloroform, 1:4‒0:1), sequentially. Fr4 was purified through Sephadex LH-20 eluted with methanol to yield five sub-fractions (SFr4-1‒SFr4-5). SFr4-2 (399.4 mg) was separated sequentially using preparative HPLC on silica gel (petroleum ether-acetone, 3:1), RP-18 (methanol-H_2_O, 4:1), and silica gel (chloroform-EtOAc, 10:1) to yield compound **6** (39.6 mg). Fr6 (1.8623 g) was separated using Sephadex LH-20 (methanol) and preparative HPLC on silica gel (chloroform-methanol, 10:1) to yield compound **10** (103 mg).

For the *A. unguis* mutant 6-20-6 (GDMCC 60337), NKA resin was added into the fermentation cultures to absorb the metabolites in the broth. Then, all the resin and mycelia were obtained together by filtration. These filtrate cakes were extracted with the mixture of methanol and acetone (2:1). The filtrate was evaporated under a vacuum to afford crude extracts (18 g). The crude extract was subjected to Sephadex LH-20 eluted with methanol to afford 11 fractions (Fr1′‒Fr11′). Fr6′ (0.26 g) was purified on an ODS column (methanol-H_2_O, 7:3 to 4:1) to give three sub-fractions (SFr6′1‒SFr6′3). SFr6′2 (79.5 mg) was separated sequentially using pTLC (petroleum ether-acetone, 4:1) to yield compound **7** (9.2 mg). Fr4′ (1.44 g) was purified by Sephadex LH-20 (methanol) to give seven sub-fractions (SFr4′1‒SFr4′7). SFr4′6 (164.2 mg) was separated using repeated pTLC (DCM-methanol, 50:1; petroleum ether-DCM, 1:3; petroleum ether-EtOAc, 3:1) to yield compound **6** (11.2 mg) and compound **8** (20.9 mg), respectively. Compound **2** (104.8 mg) was obtained from SFr4′4 (708.2 mg) by separation using Sephadex LH-20 (methanol), ODS (methanol-H_2_O, 4:1), and pTLC (petroleum ether-EtOAc, 3:1), sequentially. Compounds **9** (5.3 mg, t_R_ = 12 min), **1** (3.6 mg, t_R_ = 20 min), and **3** (26 mg, t_R_ = 44 min) were obtained from Fr8′ (0.19 g) using preparative RP-HPLC (the gradient was: 0–25 min methanol-H_2_O (3:7), flow rate: 5 mL/min; 25‒50 min, methanol-H_2_O (6:4). flow rate: 7 mL/min). Compound **4** (12 mg) was separated from Fr9′ (0.15 g) using preparative RP-HPLC (methanol-H_2_O, 4:6).

Compound **1**: Yellow amorphous solid, m.p. 166.4‒167.6 °C. HR-ESI-MS *m*/*z* 371.1137 [M − H]^−^ (calcd 371.1131 for C_20_H_19_O_7_^−^). IR ν_max_ (KBr) 3255, 1658 cm^−1^. UV-Vis (MeOH) λ_max_ (log ε) 217 (5.88), 266 (5.51), 305 (5.27) nm. ^1^H-NMR (CD_3_OD, 500 MHz), see Table 1; ^13^C-NMR (CD_3_OD, 125 MHz), see Table 1.

### 3.4. HPLC Fingerprinting

The HPLC fingerprints of *A. unguis* fermentation extracts when statically cultured under different conditions were analyzed with linear elution using Agilent Infinity II 1260. The gradient changed from 10% methanol-H_2_O to 100% methanol in 35 min, was maintained at 100% methanol for the next 5 min, then changed from 100% to 10% methanol-H_2_O within 2 min and was kept for 3 min. The flow rate was 1 mL/min. The monitoring wavelength was 254 nm. The extract samples included: (A) original *A. unguis* in potato sucrose broth (PSB) medium; (B) original *A. unguis* in PSB medium + NaBr; (C) original *A. unguis* in PSB medium + procaine; (D) original *A. unguis* in PSB medium + procaine + NaBr; (E) *A. unguis* mutant 6-20-6 in PSB medium. The concentrations of NaBr and procaine were 20 g/L and 1 mM, respectively. The fungi were cultured in 200 mL of medium in 1L flasks for 20 days under the same conditions, with the large-scale fermentation occurring as described above and the products extracted with NKA resin.

### 3.5. Antimicrobial Assay

Preliminary antimicrobial tests for compounds **1**‒**10** were carried out against pathogenetic strains, including *Pseudomonas aeruginosa* (ATCC 9027), methicillin-resistant *Staphylococcus aureus* (A7983, clinical isolate donated by Zhijun Yu in Dalian Friendship Hospital, Dalian, China), *Vibrio parahemolyticus* (ZouB, aquatic pathogen donated by Chongqing Wen in College of Fishery, Guangdong Ocean University, Zhanjiang, China), and *Candida albicans* (ATCC 10231) using the disc diffusion method [38,39]. Muller Hinton agar (MHA) and Sabouraud agar (SA) were used for antibacterial and antifungal tests, respectively. For each sample, 10 μg was added onto paper disc (diameter of 6 mm) for tests. The minimum inhibition concentrations (MICs) of the compounds were tested using microdilution methods [39,40]. Ampicillin and ketoconazole were taken as positive controls for antibacterial and antifungal tests, respectively. Solvents (MeOH and DMSO) were used as blank controls.

### 3.6. AChE Inhibition Assay

Acetylcholinesterase (AChE, from electric eels), acetythiocholine iodide (ATCh), 5,5-dithiobis-(2-nitrobenzoic acid) (DTNB), and bovine serum albumin (BSA, A1933) were purchased from Sigma-Aldrich. 100 mM PBS (pH = 7.4) was prepared with K_2_HPO_4_ and KH_2_PO_4_. In vitro AChE inhibitory activity was measured in 96-well plates using modified Ellman’s methods [41]. Compounds **1**‒**10** were dissolved in DMSO with different concentrations. Each well contained 1 μL of prepared sample, 49 μL of PBS, 10 μL of enzyme with a final concentration of 0.2 U/mL in BSA (10mM in PBS), and 20 μL of DTNB (5 mM in PBS). Then, the plates were preincubated at 37 °C for 10 min. Afterwards, 20 μL of ATCh (10 mM in PBS) was added into the well. After incubation at 37 °C for 20 min, the 96-well plates were read at 405 nm on the microplate reader, and the inhibition ratios to AChE were calculated using the following equation, where A_sample blank_ stands for the absorbance of a well without enzyme, A_sample_ stands for the absorbance of the treatment, A_contorl_ stands for the absorbance of the well without the sample, and A_blank_ stands for the absorbance of the well without the sample and enzyme. Donepezil was taken as a positive control.

$$\mathrm{Inhibition}\;\mathrm{of}\;\mathrm{AChE}\;\left(\%\right)=\frac{\left[\left(A_{\mathrm{control}}-A_{\mathrm{blank}}\right)-\left(A_{\mathrm{sample}}-A_{\mathrm{sample}\;\mathrm{blank}}\right)\right]}{\left(A_{\mathrm{control}}-A_{\mathrm{blank}}\right)}\times 100\%$$

The IC_50_ value is the concentration required to inhibit AChE activity by 50%. The inhibition rates and concentration logarithm (lnC) values were imported into the Origin 8.0 software (OriginLab Co., Ltd., Northampton, MA, USA). The cubic polynomial regression equation was used to calculate ln(IC_50_). Then, IC_50_ was calculated using Microsoft Excel.

### 3.7. DPPH Free Radical Scavenging Assay

The free radical scavenging potentials of compounds **1**‒**10** were evaluated in 96-well plates using the DPPH free radical scavenging assay described by Sharma and Bhat [42]. A total of 100 μL of the reaction mixture was composed of 50 μL of 0.16 mM DPPH (Sigma-Aldrich, St. Louis, MO, USA) in methanol and 50 μL of the test compound (in DMSO) with different concentrations (A_sample_). The reaction mixture was incubated for 30 min in darkness at room temperature. Meanwhile, 50 μL of 0.16 mM DPPH with 50 μL of DMSO was used as control (A_control_), 50 μL of methanol and 50 μL of different concentrations of the test compound in DMSO were used as the sample blanks (A_sample blank_), 50 μL methanol with 50 μL DMSO was used as the blank (A_blank_), while vitamin C was taken as a positive control. The absorbance was measured at 517 nm on the microplate reader.

$$\mathrm{Scavenging}\;\mathrm{capacity}\;\left(\%\right)=\frac{\left[\left(A_{\mathrm{control}}-A_{\mathrm{blank}}\right)-\left(A_{\mathrm{sample}}-A_{\mathrm{sample}\;\mathrm{blank}}\right)\right]}{\left(A_{\mathrm{control}}-A_{\mathrm{blank}}\right)}\times 100\%$$

The EC_50_ value is the concentration required to scavenge 50% of DPPH radicals. Likewise, the EC_50_ was calculated using Origin 8.0 plotting and cubic polynomial regression.

### 3.8. Brine Shrimp Larva Lethality Test

Compounds **1**‒**10** were evaluated for larvicidality using the brine shrimp (*Artemia salina*) larva lethality test [43]. 0.15 g of frozen eggs of brine shrimp larva (purchased from Aijia Aquarium Co., Zhanjiang, China) were activated at room temperature for 12 h. After activation, eggs were added to 1 L of artificial seawater (3% sea salt), ventilated with air for 420 mL/min, then kept at a brightness at 1000 lux and incubated at 28 °C for 24 h. After incubation, the larvae were induced to the top of the separating funnel by phototaxis, and the dead ones on bottom were discharged. Then, in each well of the 96-well plate, 1 μL of test compounds (in DMSO) with different concentrations and 99 μL of seawater containing 20‒30 live larvae were added. After incubation under light at 28 °C for 24 h, recorded the numbers of dead larvae. Finally, 10 μL of the Lucus reagent were added into each well and the total number of brine shrimp larva in the well were counted. DMSO and Hg(NO_3_)_2_ were used as blank control and positive control, respectively.

$$\mathrm{Lethality}\;\mathrm{rate}\;\left(\%\right)=\frac{\left(\mathrm{lethality}\;\mathrm{of}\;\mathrm{treatment}-\mathrm{lethality}\;\mathrm{of}\;\mathrm{blank}\;\mathrm{control}\right)}{\mathrm{survival}\;\mathrm{rate}\;\mathrm{of}\;\mathrm{blank}\;\mathrm{control}}\times 100\%$$

The brine shrimp larva LC_50_ value is the concentration required to kill 50% of the larvae. Likewise, the LC_50_ was calculated using Origin 8.0 plotting and cubic polynomial regression.

### 3.9. Molecular Docking Studies

The molecular docking studies were performed with Torpedo Californica AChE (PDB: 1DX6) retrieved from the RCSB protein data bank using the Discovery Studio (DS) 3.5 program (BIOVIA Co., Ltd., San Diego, CA, USA). For docking simulations, the original inhibitors were removed, the protein was regarded as ligand free, and water molecules were also removed from the protein structure. All polar hydrogens, a CHAEMm force field, and a Momany-Rone charge were added into the protein. The 2D structure of **1** and **9** were drawn with ChemDraw Ultra 7.0 (PerkinElmer Co., Ltd., Waltham, MA, USA), converted into a 3D pdb format, and their minimized energy was obtained by using DS 3.5. The active center and active pocket are identified by DS 3.5 software. The X, Y, and Z of active centers were 3.66, 65.90, and 64.11, respectively. The radius of active centers is 20.0 Å. Molecular docking of compounds **1** and **9** into the AChE proteins binding sites were performed using the CDOCKER protocol. The conformation with the highest negative CDOCKER interaction energy was picked out for further analysis. Molecular graphics and analyses were performed with DS 3.5 [29,44].

## 4. Conclusions

In summary, in the present work, 10 natural compounds—including a new despidone (**1**) together with eight known despidones (**2**‒**9**), and a cyclic peptide (**10**)—were obtained from a marine fungus *A. unguis* under chemical induction and from its plasma-induced mutant. The antibacterial and/or antifungal effects of **1**, **5**, **7**, **8**, and **9** suggest their potential as lead compounds for antibiotic discovery. Compounds **3**, **4**, and **5** could be used for the development of pesticides for their larvicidal activity. The docking study shows that the negative CDOCKER interaction energy between AChE and **9** is higher than **1**, validating the better in vitro inhibitory activity of **9** compare to **1**. To the best of our knowledge, compound **9** is the first reported depsidone from marine fungi that is an AChE inhibitor. The HPLC fingerprint analysis indicates that chemical induction and plasma mutagenesis are able to effectively influence the secondary metabolism of marine fungi. Furthermore, NaBr and procaine (a DNA methyltransferase inhibitor) showed synergistic effects in chemical induction, including enhancing the integration of bromine into molecules. The analysis of plausible biosynthetic pathways implies that chemical induction and plasma mutagenesis can regulate key steps in depsidone biosynthesis. They can be used as tools to increase the chemodiversity of fungi and obtain useful natural products.

## 5. Patents

Zhang, Y.; Yang, W.C.; Bao, H.Y.; Liu, Y.Y.; Nie, Y.Y.; Yang, J.M.; Hong, P.Z.; Qian, Z.J.; Song, C.; Liang, J.Y.; Li, Z.P. A new depsidone, its preparation method and application. China Patent Database. Application Number: 201810639532.8.

Zhang, Y.; Yang, W.C.; Bao, H.Y.; Liu, Y.Y.; Nie, Y.Y.; Yang, J.M.; Hong, P.Z.; Qian, Z.J.; Song, C.; Liang, J.Y.; Li, Z.P. A marine fungus mutant strain *Aspergillus unguis* 6-20-6. China Patent Database. Application Number: 201810639846.8.

Zhang, Y.; Yang, W.C.; Bao, H.Y.; Liu, Y.Y.; Nie, Y.Y.; Yang, J.M.; Hong, P.Z.; Qian, Z.J.; Song, C.; Liang, J.Y.; Li, Z.P. The Application of a depsidone. China Patent Database. Application Number: 201810639845.3.

## Figures and Tables

**Table 1:** NMR data for **1** measured in CD_3_OD.

Position	*δ*c ^a^, Type	*δ*_H_^b^, Mult (*J* in Hz)	Position	*Δ*c ^a^, Type	*δ*_H_^b^, Mult (*J* in Hz)
1	104.5, C		3′	116.9, C	
2	144.7, C		4′	161.8, C	
3	113.5, CH	6.28, dd, (2.0, 0.5)	5′	114.3, CH	6.25, s
4	166.9, C		6′	148.7, C	
5	102.2, CH	6.19, d, (2.4)	7′	141.1, C	
6	165.9, C		8′	120.6, CH	5.34, dq, (6.8, 1.3)
7	171.3, C		9′	14.0, CH_3_	1.70, dd, (6.8, 0.8)
8	24.6, CH_3_	2.59, s	10′	18.8, CH_3_	1.92, s
1′	116.7, C		11′	9.6, CH_3_	2.01, s
2′	151.3, C		12′	175.2, C	

^a 13^C-NMR recorded in CD_3_OD at 125 MHz; ^b 1^H-NMR recorded in CD_3_OD at 500 MHz.

**Table 2:** Antimicrobial activities of compounds **1**‒**10**, measured using the paper disk method.

	Strains	Zone of Inhibition (mm) ^#^			
Compounds		*P. aeruginosa*	MRSA	*V. parahemolyticus* *	*C. albicans*
**1**		7.7 ± 0.6	12.0 ± 1.0	9.3 ± 0.6	8.3 ± 0.6
**2**		-	-	8.0 ± 0.0	6.0 ± 0.5
**3**		12.2 ± 0.3	-	-	12.1 ± 0.2
**4**		9.5 ± 0.6	9.0 ± 0.0	-	9.0 ± 0.0
**5**		11.9 ± 0.2	14.5 ± 0.6	6.3 ± 0.6	7.5 ± 0.6
**6**		8.7 ± 2.1	7.0 ± 0.0	7.0 ± 0.0	8.0 ± 0.5
**7**		10.0 ± 0.0	13.7 ± 2.3	9.7 ± 0.6	7.9 ± 0.2
**8**		11.1 ± 0.2	17.0 ± 0.0	8.3 ± 0.6	10.0 ± 0.0
**9**		16.0 ± 0.0	17.7 ± 0.6	8.3 ± 0.6	15.5 ± 2.2
**10**		-	-	9.0 ± 0.0	6.0 ± 0.5
Ampicillin		12.0 ± 1.4	14.0 ± 0.0	9.0 ± 0.0	-
Ketoconazole		-	-	-	22.0 ± 0.0

^#^: mean ± SD of three replicates; -: no or very weak activity; *: incomplete inhibition.

**Table 3:** Antimicrobial activities of compounds **1**‒**10** tested by microdilution method.

	Strains	MIC (μM)			
Compounds		*P. aeruginosa*	MRSA	*V. parahemolyticus*	*C. albicans*
**1**		102.4	51.2	>102.4	12.8
**2**		-	-	>102.4	-
**3**		>102.4	-	-	>102.4
**4**		>102.4	102.4	-	>102.4
**5**		6.4	25.6	>102.4	>102.4
**6**		102.4	>102.4	>102.4	>102.4
**7**		25.6	>102.4	>102.4	>102.4
**8**		51.2	25.6	>102.4	>102.4
**9**		102.4	12.8	>102.4	6.4
**10**		-	-	>102.4	-
Ampicillin		1.6	6.4	102.4	-
Ketoconazole		-	-	-	1.9

-: not tested.

**Table 4:** AChE inhibitory activity, DPPH free radical scavenging activity, and larvicidality assay of compounds **1**‒**10**.

Compounds	AChE Inhibitory Activity (IC_50_, μM)	DPPH Scavenging Activity (EC_50_, μM)	Larvicidal Activity (LC_50_, μM)
**1**	>102.4	>102.4	>102.4
**2**	>102.4	>102.4	>102.4
**3**	>102.4	>102.4	12.8
**4**	>102.4	>102.4	4.5
**5**	>102.4	>102.4	12.8
**6**	>102.4	>102.4	>102.4
**7**	>102.4	>102.4	72
**8**	>102.4	>102.4	75.9
**9**	56.8	>102.4	51.2
**10**	>102.4	>102.4	>102.4
Donepezil	0.3	-	-
Vitamin C	-	31.8	-
Hg(NO_3_)_2_	-	-	77.0

-: not tested.

## Supplementary Materials

The following are available online. Figure S1: 500 MHz for 1H-NMR Spectrum of **1** in CD3OD; Figure S2: 125 MHz for 13C-NMR Spectrum of **1** in CD3OD; Figure S3: 500 MHz for 1H–1H COSY Spectrum of **1** in CD3OD; Figure S4: 500 MHz for HSQC Spectrum of **1** in CD3OD; Figure S5: 500 MHz for HMBC Spectrum of **1** in CD3OD; Figure S6: 125 MHz for Dept 135 Spectrum of **1** in CD3OD; Figure S7: HR-ESI-MS of 1; Figure S8: Optimized structure of **1**; Figure S9: Optimized structure of **9**.
