# Supplementary material for: Depsidone Derivatives and a Cyclopeptide Produced by Marine Fungus Aspergillus unguis under Chemical Induction and by Its Plasma Induced Mutant
Source: Molecules. 2018 Sep 3;23(9):2245. doi: 10.3390/molecules23092245 (PMC6225302; doi:10.3390/molecules23092245)
Supplement: Supplementary file 1 [file molecules-23-02245-s001.zip › molecules-351721-SI.pdf]

Supplementary Materials: Depsidone Derivatives and a Cyclopeptide Produced by Marine Fungus *Aspergillus unguis* under Chemical Induction and by Its Plasma Induced Mutant

Wen-Cong Yang, Hai-Yan Bao, Ya-Yue Liu, Ying-Ying Nie, Jing-Ming Yang, Peng-Zhi Hong, and Yi Zhang

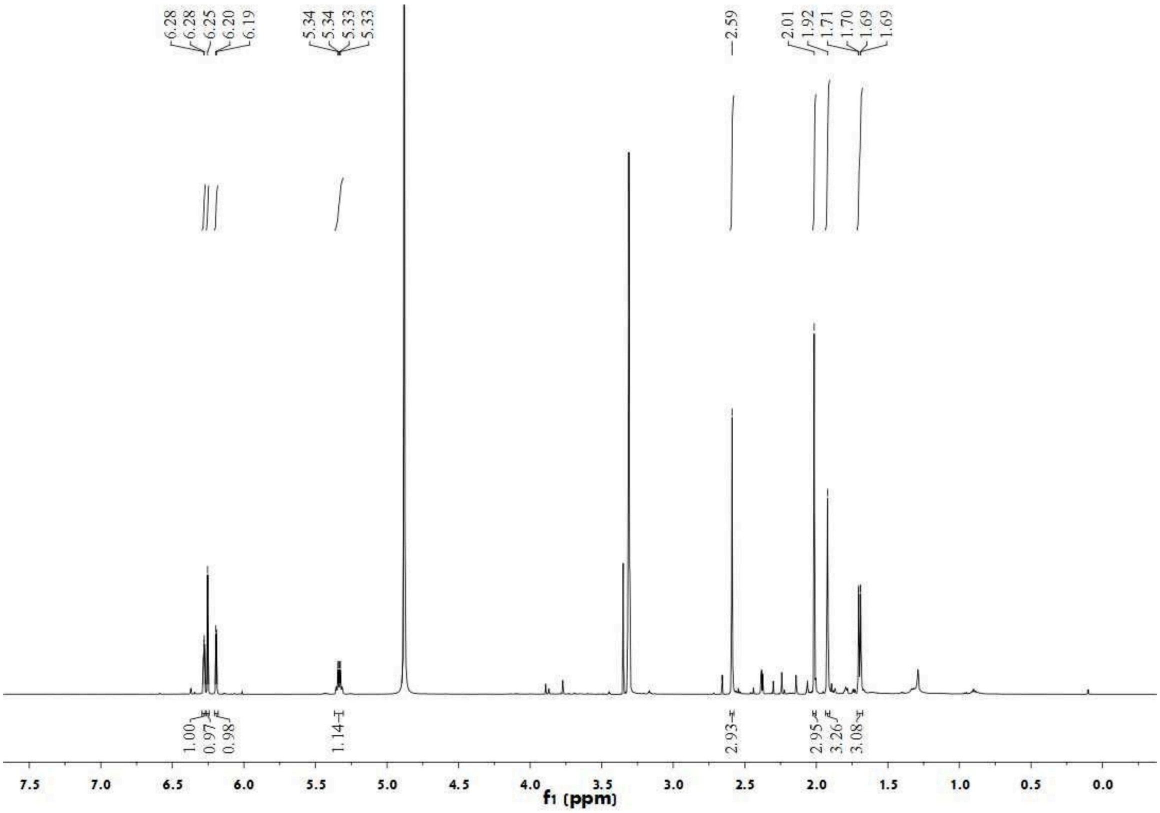

Figure S1. 500 MHz for ^{1}H NMR Spectrum of **1** in CD_3OD.

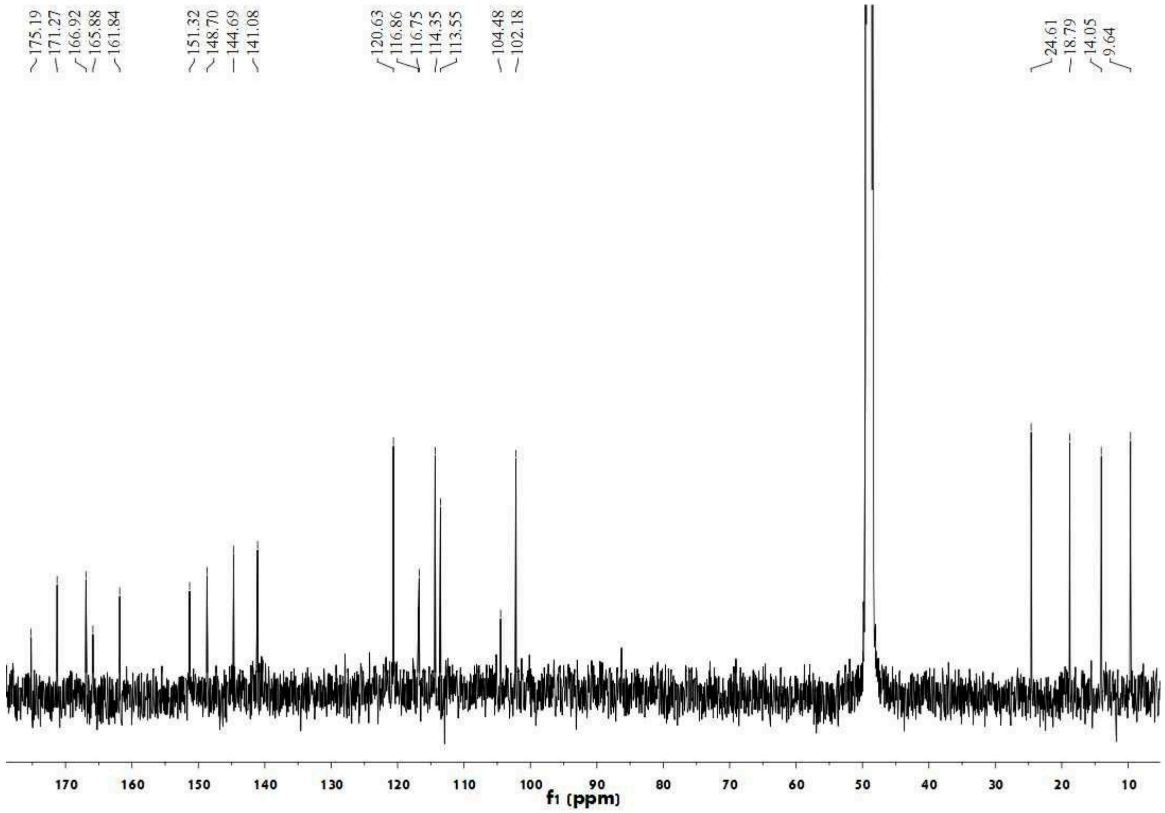

Figure S2. 125 MHz for ^{13}C NMR Spectrum of **1** in CD_3OD.

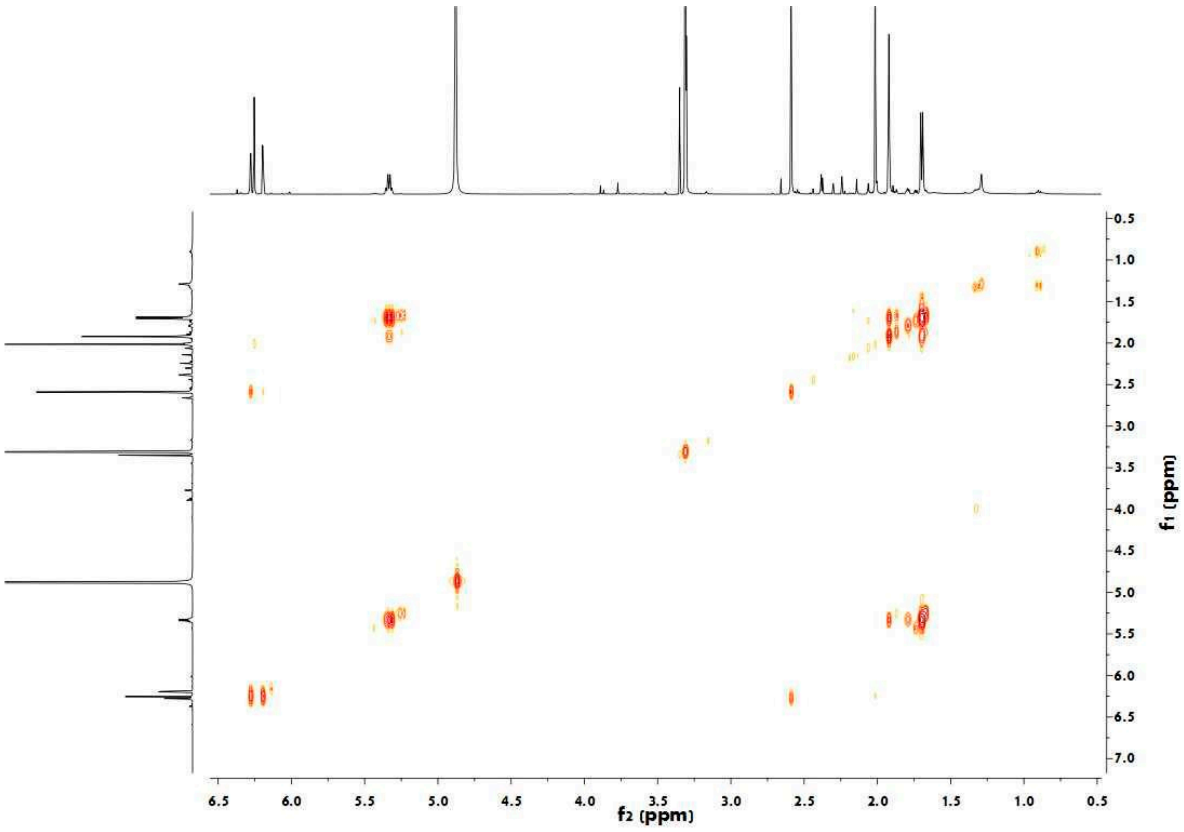

Figure S3. 500 MHz for ^{1}H-^{1}H COSY Spectrum of **1** in CD_3OD.

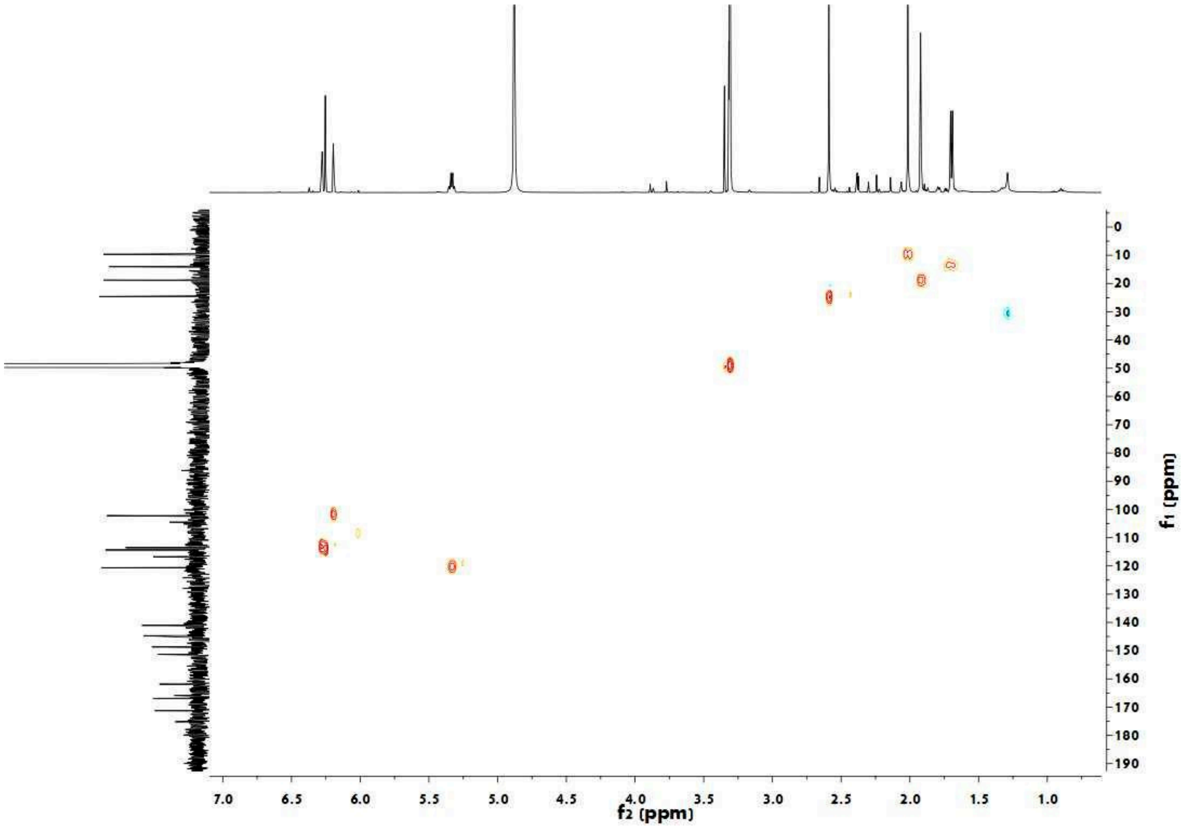

Figure S4. 500 MHz for HSQC Spectrum of **1** in CD_3OD.

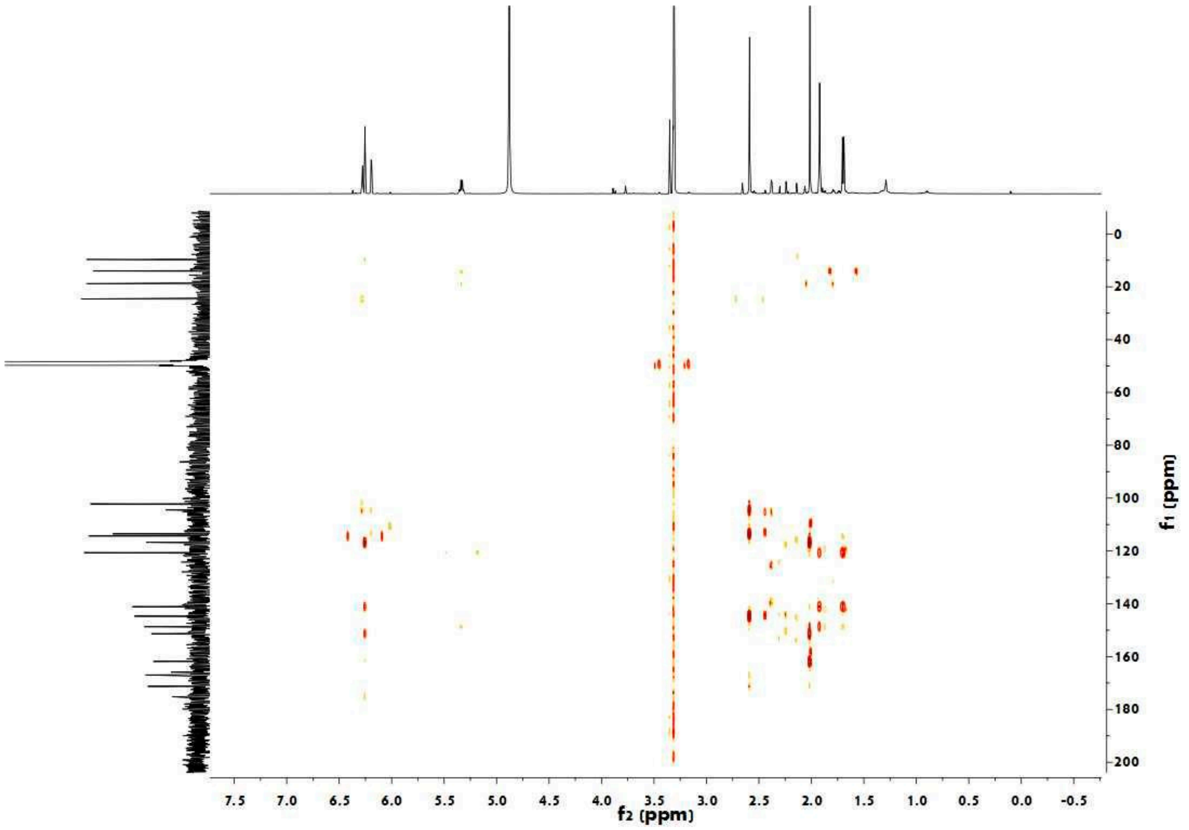

Figure S5. 500 MHz for HMBC Spectrum of **1** in CD_3OD.

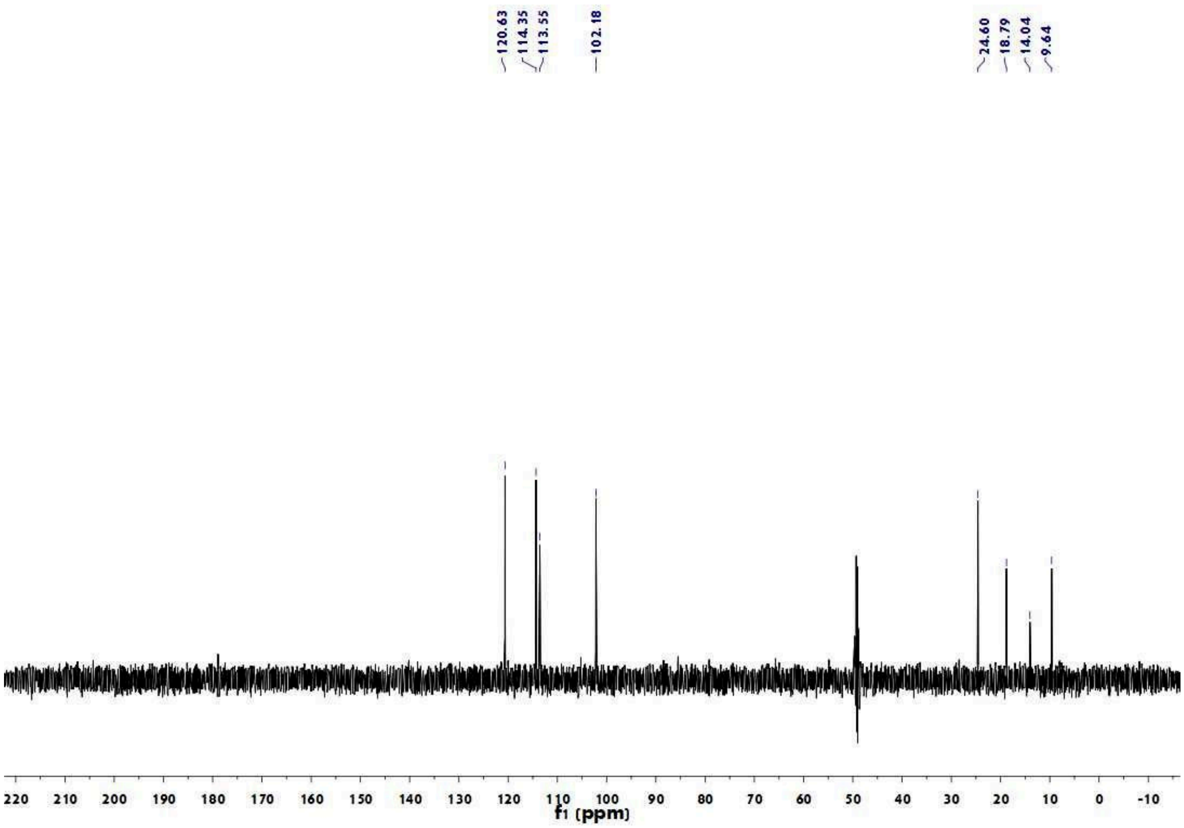

Figure S6. 125 MHz for Dept 135 Spectrum of **1** in CD_3OD.

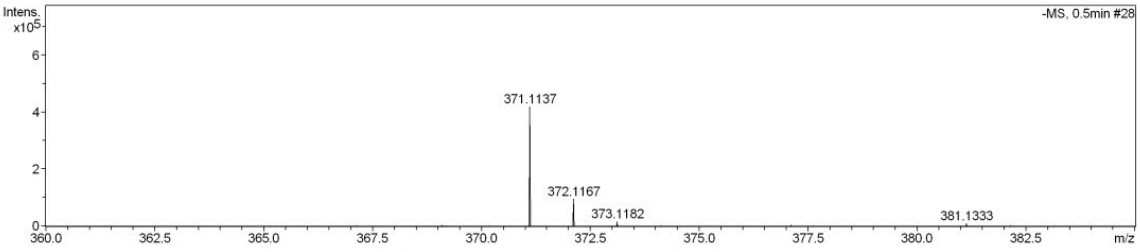

Meas. m/z	#	Ion Formula	Score	m/z	err [ppm]	err [mDa]	mSigma	rdb	e⁻ Conf	N-Rule
371.1137	1	C20H19O7	100.00	371.1136	-0.2	-0.1	4.9	11.5	even	ok

Figure S7. HR-ESI-MS of **1**.
